# Dietary Behavior Assessments in Children—A Mixed-Method Research Exploring the Perspective of Pediatric Dieticians on Innovative Technologies

**DOI:** 10.1016/j.cdnut.2023.100091

**Published:** 2023-04-26

**Authors:** Femke J. de Gooijer, Marlou Lasschuijt, Renate F. Wit, Edith JM. Feskens, Elske M. Brouwer-Brolsma, Guido Camps

**Affiliations:** 1 Division of Human Nutrition and Health, Department Agrotechnology and Food Sciences, Wageningen University and Research, Wageningen, The Netherlands; 2 OnePlanet Research Centre, Wageningen, The Netherlands

**Keywords:** qualitative and quantitative research, pediatric dietetics, children, dietary behavior assessment, technology

## Abstract

**Background:**

Assessing dietary intake and eating behavior in children is challenging, owing to children’s undeveloped food knowledge and perception of portion sizes. Additionally, caregivers cannot always provide complete surrogate information. Consequently, validated dietary behavior assessment methods for children are limited, but technological innovations offer opportunities for the development of new tools. One of the first steps in the developmental process of a newly developed pediatric dietary assessment tool includes an alignment of the needs and preferences of pediatric dieticians (PDs) as potential users.

**Objectives:**

To explore opinions of Dutch PDs about traditional dietary behavior assessment methods for children and potential technological innovations to replace or support traditional methods.

**Methods:**

Ten PDs participated in semistructured interviews (total of 7.5 h) based on 2 theoretical frameworks, and data saturation was reached after the seventh interview. Interview transcripts were inductively coded in an iterative process, and overarching themes and domains were identified. Interview data were then used as input for an extensive online survey completed by 31 PDs who were not involved in the initial interview rounds.

**Results:**

PDs discussed their perspective on dietary behavior assessments in 4 domains: traditional methods, technological methods, future methods, and external influences on these methods. Generally, PDs felt that traditional methods supported them in reaching their desired goals. However, the time needed to obtain a comprehensive overview of dietary intake behavior and the reliability of conventional methods were mentioned as limitations. For future technologies, PDs mention *the ease of use* and *engaging in children* as opportunities.

**Conclusions:**

PDs have a positive attitude toward the use of technology for dietary behavior assessments. Further development of assessment technologies should be tailored to the needs of children in different care situations and age categories to increase its usability among children, their caregivers, and dietician. *Curr Dev Nutr* 2023;xx:xx.

## Introduction

Global obesity rates among 5- to 19-y-old children increased strongly during the past decades, that is, being 4% in 1976 and 18% in 2019 [1]. In 2020, >39 million children younger than 5 y were overweight or obese according to the WHO. Dietary behavior assessment (DBA), the assessment of dietary intake and eating behaviors, plays a pivotal role in understanding, preventing, and treating diet-related diseases such as childhood obesity and associated metabolic disorders. In nutritional research, DBA is fundamental to study the origin of nutrition-related conditions, monitor trends, and plan or evaluate policies and intervention strategies. In clinical settings, DBAs are used by health professionals to evaluate diet adequacy and design personalized intervention programs.

Commonly used dietary assessment methods include self-report food records, diet histories, recalls, and food frequency questionnaires [2]. Assessment methods request a certain level of cognition, literacy, and memory to complete and hence, in case of young children, the parent or caregiver serves as a proxy reporter [3]. These methods are burdensome and prone to error [4,5], even more when multiple proxy reporters are involved in the case of school-going children [6]. As a child develops cognitively, around 10 y of age, they are considered capable of recording intake themselves [3]. However, procedures are often perceived as tedious [7], and challenges remain including difficulties in identifying or recalling different foods and beverages, intrusion of foods and beverages, estimating portion sizes, and inadequately registering intake due to limited vocabulary [[7], [8], [9]].

Recent technological advancements may help to overcome some of the current limitations of traditional dietary assessment methods [[10], [11], [12]]. The transition from paper-based to digital methods lower the burden for the dietician, parent, and child, for example, by adding image recordings to clarify consumed foods, prompting notifications to remind the user to complete the assessment, and linking the tool to a database to allow the user to search for specific food items. Technological methods are also considered to be more appealing and engaging compared with paper-based methods [10,13], which may result in higher completion rates and eventually increase the accuracy of the assessment as well. However, validated technologies tailored to the needs and understanding of children are lacking, especially those tailored to the Dutch language and eating cultures. Such innovations would be valuable for pediatric dieticians (PDs). PDs are nutritional health care providers who are specialized in the treatment of children from newborns to adolescents, which makes their practical knowledge on the use of assessment approaches in children of valuable input to create feasible, useful, and user-friendly applications. Besides, codesign and collective creativity across the entire design process [14], between developers and PDs is also critical for user acceptance [15] and successful development and implementation in a health care setting [16].

However, little is known about the opinion of PDs on the use of technology for DBAs. Several reviews have evaluated the use of technological methods for DBAs in children but mainly focused on the validity of the presented methods and provide a primarily objective perspective [10,13]. An interdisciplinary perspective study with health care providers in primary care suggested that there is a strong potential for using electronic dietary assessment tools in their daily practice, although this study did not provide information about the use of technologies in pediatric care [17]. It is important to understand what PDs consider facilitating characteristics of current assessment methods and what they foresee as challenges and benefits of incorporating technology in their field to give more guidance in the development of assessment technologies for children and to ensure in good user acceptance among PDs. Therefore, this study aimed to examine the perspective of PDs on traditional DBA methods for children and the potential of technological innovations to replace or support traditional methods.

## Methods

A mixed-methods study was conducted to gain insight into the thoughts, experiences, and preconceptions of PDs on the use of technology for dietary assessments in children, including semistructured interviews (*n* = 10) and an online survey (*n* = 31). The Consolidated Criteria for Reporting Qualitative Studies (COREQ) [18] and the Checklist For Reporting Results of Internet E-Surveys (CHERRIES) [19] were used to ensure adequate reporting on the qualitative and quantitative procedures in this study.

### Participant recruitment

For the interviews, a call for Dutch PDs was shared utilizing LinkedIn and the newsletter of the Dutch Network of Paediatric Dieticians, a collaboration of qualified pediatric dieticians working in all health sectors throughout The Netherlands. For the interviews, interested PDs contacted us by e-mail and received more detailed information about the study aim and procedures, answers to questions, and time slot for the interview. To establish good coverage of the varieties of PDs in the field, we recruited PDs from different health care facilities and various age groups. PDs were eligible for participation when they: *1*) assessed dietary behavior in children at least once a week, and *2*) provided informed consent, including the willingness to be recorded during the interview. Data saturation was assumed to be acquired within 6–12 interviews [20], which in this study appeared to be reached after 10 interviews [21] when no new codes appeared in the last 3 interviews. For the survey, the link to the survey was shared utilizing the interviewed PDs, LinkedIn, and the newsletter of the Dutch Network of Paediatric Dieticians. Dieticians who participated in the interview could not complete the survey. A sample size of 30 was considered adequate [22] to represent the population of 183 PDs connected to the Dutch Network of Paediatric Dieticians in The Netherlands. Participation in the interviews and the survey was completely voluntary and not financially compensated. All participating dieticians signed informed consent indicating their approval to take part in the study and allow the survey data to be used. The study was conducted adhering to the ethical standards and guidelines of Wageningen University and Research.

### Qualitative data collection

For the semistructured interviews, an interview guide was developed based on a combination of 2 theoretical frameworks (Information–motivation–behavior model [23], User Experience Honeycomb model [24]). This guide was tested in a pilot interview and revised after assessing the preliminary results. The overall interview was divided into 3 parts: *1*) identification of the personal context, *2*) evaluation of currently used (technological) assessment methods, and *3*) perception of future (technological) assessment methods (Supplemental Table 1). One week before the interviews, all participants received information about the procedure and the informed consent form. Due to the prevailing COVID-19 regulations at the time, all interviews were held via Microsoft (MS) Teams or other video calling platforms (if necessary) and were conducted by the primary researcher. Synchronous video-aided online interviewing, such as interviews via MS Teams, provide an equal authenticity level compared with face-to-face interviews due to access to verbal and nonverbal cues [25]. The relative anonymity of online interviews may even increase authenticity and therefore, using MS Teams for the interviews was not considered problematic. At the start of each interview, the participants were asked if they had any questions about the procedure. During the interviews, in addition to the interview guide, probing questions (for example, “can you elaborate on that?,” “what do you mean by X?”) were used to gain more information from the participants. In the third part of the interview, examples of technological developments were given by the interviewer to stimulate the conversation. The semistructured interviews lasted ∼45 min (range = 35–58 min) and were conducted between July and September 2021.

### Quantitative data collection

The online survey was based on the findings from the interviews, the theoretical framework, and, to quantify user experience, the short version of the user experience questionnaire (UEQ) [26]. The short UEQ was designed to obtain the impression of users toward the usability of products in terms of hedonic quality aspects (*supportive*, *easy*, *efficient*, and *clear*) and pragmatic quality aspects (*exiting*, *interesting*, *inventive*, and *leading-edge*). The questionnaire consists of 4 hedonic and 4 pragmatic pairs of qualities with opposite meaning (for example, *confusing* and *clear*) that are rated on a 7-point Likert scale (−3 fully agree with negative term, +3 fully agree with positive term). Two pairs, *reliable/unreliable* and *accurate/inaccurate*, were added to the 8 pairs due to the frequent appearance of these terms in the interviews. Answer options in multiple answer questions were also based on the answers given in the interview. The questionnaire was discussed in the research team to agree upon the final version. This final version consisted of 33–59 questions, depending on the number of DBA methods used, as indicated by the respondent. Before the survey, all respondents were asked to confirm performing DBAs on children at least once a week and asked for informed consent. Internet Protocol (IP) addresses were used to identify potential duplicate entries. On average, the survey was completed in 37 min, and 84% of the respondents completed the survey within 25 min. The survey was published using Qualtrics [27] and was available online during November and December 2021.

### Qualitative data analysis

All audio-recorded interviews were transcribed using Amberscript [28], after which the transcripts were corrected by 2 researchers (FG and RW). To thematically organize the interviews, the interview transcripts were coded using QDA Miner [29] adopting an iterative open-coding strategy. This strategy implied that 2 researchers (FG and RW) independently coded the first 3 interviews, after which a first coding template was generated by comparing and merging both coding schemes. Using this first coding template, the next 3 interviews were coded, again by both researchers independently, and subsequently, both codes were compared and merged into an updated coding template. This procedure was repeated until all the interviews were finished. The obtained codes were sorted and categorized by 4 researchers independently (FG, RW, ZH, and ML). The evolved categories were discussed within the research team to agree upon a final coding tree (Supplemental Figure 1), themes, and domains. Finally, illustrative quotes were selected and translated from Dutch to English.

### Quantitative data analysis

Data analysis was performed using RStudio [30]. Only data of respondents who completed the survey were included after which descriptive analysis was performed. For categorical data, frequency distributions were calculated. Fisher’s exact test was used to identify differences between age groups, experience, and health care settings for frequency distributions. UEQ scores were interpreted as follows: 0.8 and 0.8 represent a neutral evaluation, above 0.8 a positive evaluation and below −0.8 a negative evaluation of usability [31]. The comparison of UEQ scores was conducted using a paired 2-sided *t*-test. Only data from respondents who assessed ≥1 technical and ≥1 traditional method were included. Scores were averaged when multiple methods were evaluated.

## Results

In total, 10 Dutch PDs participated in the semistructured interviews. Forty-one PDs initially consented to the online survey of which 10 PDs were excluded due to incomplete survey responses (Table 1). The mean age of the participants in the interview was 45.1 (SD = 14.6) y with 17.1 (SD = 12.1) y of experience in the field of pediatric dietetics. The mean age of the participants in the online survey was 44.1 (SD = 12.7) y with 13.1 (SD = 11.4) y of experience. All participating PDs were women. Three PDs participating in the interviews were employed in primary health care, 6 in secondary health care, and 1 was employed in both. In the online survey, 13 PDs were employed in primary health care settings, 11 in secondary health care, and 1 was employed in both. Additionally, 6 PDs responding to the survey were in tertiary health care and 1 PD was employed in industry.

**TABLE 1:** Descriptive characteristics of participating pediatric dieticians in the interviews (*n* = 10) and in the online survey (*n* = 31)

Characteristics	Mean ± SD, *n* (%), Min–max	
	Interviews (*n* = 10)	Online survey (*n* = 31)
Age, y	45.1 ± 14.6	44.1 ± 12.7
Range	25–63	23–64
In practice, y	17.1 ± 12.1	13.1 ± 11.4
Range	1–33	1–38
Gender		
Female	10 (100%)	24 (77%)
Unknown	—	7 (23%)
Provided care		
Primary care	3 (30%)	13 (42%)
Secondary care	5 (50%)	11 (36%)
Tertiary care	1 (10%)	5 (16%)
Other^1^	1 (10%)	2 (6%)

1 Other care provided was pediatric dieticians working in health care industries or working in both primary and secondary health care.

### Qualitative results

The major themes from the interviews fell into the following 4 domains: *Traditional methods*, *current technologies, external influences, and future methods.*

#### Domain 1: traditional methods

The domain of *traditional methods* explores the role of DBA in current pediatric dietetics and elaborates on the use of traditional methods as well as factors defining the choice for and success of DBA methods. Themes and supporting quotes are presented in Table 2. The most common method used by the interviewed PDs to assess dietary behavior in children was the oral dietary history method and paper-based food records. Both methods were used to identify possible improvements in dietary patterns, to identify the caloric or nutritional needs of patients, or to monitor the intake of specific nutrients. Diet history was specifically used to identify eating behaviors (quote 1.1 in Table 2), and PDs choose to use a food record as preparation before a consult, to provide insight for parents and children or to collect more detailed or realistic information when the diet history was not sufficient (quote 1.2 in Table 2). The majority of the interviewed PDs felt that traditional assessment methods were sufficient in helping them reach the desired goal but also various limitations were raised, including duration, accuracy, and reliability.

**TABLE 2:** Emerging themes and illustrative quotes relating to traditional methods for dietary behavior assessment (domain 1) discussed by Dutch pediatric dieticians

Theme	Illustrative quotes	
	1.1	“The goal of using an oral dietary history is mainly to get an idea of what and how much are they eating, and I also get an idea on how it is done, do they eat at a table, at the couch, are they together or alone.”
	1.2	“Usually I use the dietary history, often you can get a lot of things out of that. But especially if there are still many questions or, for example, nutritional advice and the results do not add up, then I want to know a bit more and use a record.”
Duration	1.3	“I try to keep the assessment under 10 minutes, but I think it is a waste of time because if the food record was filled in, you are finished way sooner.”
	1.4	“In primary care, only 3 hours of dietetics are reimbursed, whereas in secondary care it is unlimited. So, yes, if I spend my first hour on questioning what somebody eats, then that is no problem. But in primary care, this does matter as you will only have 2 hours left.”
Accuracy	1.5	“I think that if you do a diet history, you get an average day, but this is not how it is in reality. And food records are not filled in properly.”
Reliability	1.6	“Yes, with children who suffer from a metabolic disease and you need to know everything accurately to the milligram, the parents are often highly motivated to write everything down to the exact gram. For example, they are only allowed 4.3 grams of protein a day. Then you have to do the math.”

##### Duration

The duration of traditional dietary assessment methods was frequently mentioned as a limitation, especially when the patient was not adequately prepared (quote 1.3 in Table 2). Moreover, the time needed to process the findings after a consultation was considered burdensome. Time was particularly an issue for primary care PDs, as only 3 h of dietetic consults can be reimbursed by primary care in The Netherlands (quote 1.4 in Table 2).

##### Accuracy

Traditional dietary behavior assessment methods lack accuracy (quote 1.5 in Table 2). However, the need for a certain level of accuracy highly depends on the purpose of the assessment: “For overweight, I think it is less important because it does not depend on the exact number of calories.” In other situations, high accuracy is required: “It is important because it is the base of your advice. … for children with diabetes need to consume a certain amount of carbohydrates, I want to know the exact grams and how they calculate it.”

##### Reliability

As with the level of accuracy, reliability is also a factor that depends on the purpose of the assessment. In general, PDs say they want the assessment to be “As reliable as possible. But you know that there is underreporting. And I think that will always be the case, how hard they try to fill it in correctly.” For patients with the more severe diseases, reliability can be very important and also related to the motivation of the caretaker (quote 1.6 in Table 2). Although children have difficulties recalling consumed foods, their honesty improves reliability: “Sometimes it says something like ‘bowl of carrots’ and then I ask, ‘did you not have any sweets after school?’ ‘Yes! I always get sweets after school!’”

#### Domain 2: current technologies

The domain *current technologies* are closely linked to the domain *traditional methods* and includes current use of technological DBA methods by PDs and the impression of PDs toward using these methods with corresponding advantages and limitations. Themes and supporting quotes are presented in Table 3. The majority of the interviewed PDs already had some experience with dietary behavior assessment technology. When asked why some of the PDs did not use any technologies for dietary behavior assessment, they answered that this was because of a lack of knowledge or convenience to use what is known (quote 2.1 in Table 3).

**TABLE 3:** Emerging themes and illustrative quotes relating to current technologies for dietary behavior assessment (domain 2) discussed by Dutch pediatric dieticians

Theme	Illustrative quotes	
	2.1	“Unfamiliarity, you have to spend some extra time on it to see if it works. In practice you keep going with what you are used to, and you do not seek for something new.”
Advantages	2.2	“Currently, I use the digital food record with another goal, I want people to get insight themselves. … A paper-based record that they send to me that is more for me to get insight.”
	2.3	“What is the atmosphere at the dining table? What are the interactions? … What is the attitude of the child? ... How is the intake and how fast do they eat? This kind of things all show in 1 little video recording.”
Limitations	2.4	“You have to be quite smart to use these apps. You have to know what search terms to use, for example, when you’re looking for whole wheat bread, you should not look for ‘whole wheat’ but for ‘bread’ and then look for the whole wheat version.”
	2.5	“After you filled it in, you immediately see calories. You do not see a green dot or something, but you immediately see a number. … For parents that can be clarifying but for children I don’t think that is a good idea.”

##### Advantages

PDs choose to use digital food records to get a more detailed information on, for example, vitamin and mineral intake or to provide patients with insights into their own dietary intake (quote 2.2 in Table 3). PDs who used digital food records, mentioned its ease of use and efficiency as an advantage: “ ... if it is delivered to me digitally, of course, then I do not have to retype everything, then you can go straight to the results.” Cameras on mobile phones were used to take pictures of meals to support the recall process or to clarify specific food products during consultations or to record videos to study eating behaviors at home mealtimes (quote 2.3 in Table 3).

##### Limitations

A lack of usability of current technologies was highlighted, especially for children. Current applications are mentioned to be too complicated (quote 2.4 in Table 3), inappropriate for children (quote 2.5 in Table 3), or not exciting enough: “I think the strength of the ‘Mijn Eetmeter’ is that it is governmental and super decent. But at the moment that’s also its weakness ... people find it dull.”

#### Domain 3: external influences

Four external factors were identified that influence dietary behavior assessments. These were the age and autonomy of the children, the role of their caregivers, cultural background, and the role of the PDs themselves. The theme *external influences* addresses external factors that affect the success of dietary behavior assessment methods for children. Themes and supporting quotes are presented in Table 4.

**TABLE 4:** Emerging themes and illustrative quotes relating to external factors influencing dietary behavior assessments (domain 3) discussed by Dutch pediatric dieticians

Theme	Illustrative quotes	
Age and autonomy	3.1	“I think children from about the age of 6 are able to tell a few things quite well, but that is definitely not complete, parents really need to complete a lot. But you can have a conversation with them about it.”
	3.2	“At the end of primary school, I tell a kid of 10 years old: ‘well, you are 10, you are the one who decides what you put in your mouth. So, I want to hear from you how we should do things.’”
	3.3	“I think from the age of 10 you will have the chance that it works, but then still, I look at the child and I consult the parents if they can do it together.”
	3.4	“Sometimes, children buy stuff themselves, so if they eat much more, it is hard to notice.”
	3.5	“Of course, we are in the hospital, and if there is 1 thing you don’t want as an adolescent, it is to be preoccupied with your illness.”
Parents and caregivers	3.6	“Because most adolescents don’t say everything when mom and dad are around. Especially things like sweets and alcohol.”
Cultural background	3.7	“The Dutch language, but also reading and writing. Or in general, how literate or how highly educated people are, that is often an important factor. In particular the parents in this case. Also, the children, but then it is mainly the age of the child ... however, it is also important to know whether the child is in special education or in public school, and I try to match that, it is how you ask for the information and how you give it back.”
	3.8	“80% of the children I see are foreign-born or non-Dutch. I don’t think they will be open to it unless they are highly educated.”
Pediatric dietician	3.9	“I want there to be a relationship of trust. They need to feel that I would never criticize them.”
	3.10	“Sometimes you see this in the consulting room, and you think, oh yes. If a child looked 3 times, the mother would reach into her bag for a snack. You can sense these little things.”

##### Age and autonomy

Age and, closely related, autonomy play an important role in the assessment approach of PDs. Although PDs indicated that there were large differences in autonomy between children with similar ages, 3 age limits could be identified to which the assessment approaches were adapted, that is, around 6 y old (children can be involved in the conversation; quote 3.1 in Table 4), 10–11 y old (children become the main interlocutors; quote 3.2 in Table 4 and children can start, although guided, keeping their own food record; quote 3.3 in Table 4), and 14 y old (food record can completely be filled in independently): “When they are 14 or so. Before that age, I notice that children write down ‘sandwich’ or ‘bread,’ and I think: yes, that’s nice, but that doesn’t help me. … I think that will be a good age to ask them to do it themselves.” However, the autonomy of adolescents comes with other challenges (quotes 3.4 and 3.5 in Table 5).

**TABLE 5:** Emerging themes and illustrative quotes relating to future methods for dietary behavior assessment (domain 4) discussed by Dutch pediatric dieticians

Theme	Illustrative quote	
Accessible	4.1	“I can imagine that with a digital tool you might ask a bit more questions. That if someone only fills in butter, the tool will ask: ‘hey but what kind of butter?’ Or ‘was there also something to drink?’”
	4.2	“The 3-year-olds already get the discarded mobile phones from their parents. I have hardly any children who don’t have 1, even the families who don’t have a penny to their name have an iPhone in their bag.”
Stimulating	4.3	“That children can serve themselves until the plate turns green, for example, and if it turns orange, then it is a little too much. … In this way they learn: ‘Okay, this is the right portion for me’ but they can decide for themselves how much will be on the plate”
	4.4	“Especially with overweight, you want positive feedback. For example, a sports watch can tell you if you move 500 calories I am allowed to eat extra and still lose weight. … it stimulates to move because you get direct feedback”
Functional	4.5	“New technologies should be user friendly and practical. Details about a bottle being empty in 10 or 30 sips, I will not use. I want concrete and practical information that is not as detailed as scientific information”
	4.6	“I see some children who spend more than 45 minutes at the dining table before something is finished. Is most of it eaten at the beginning or at the end?”
Challenges	4.7	“Now we give them a folder with exercises to do at home and you see that after a while they forget to bring this folder. This will be the same with an app, you can ignore messages and pop-ups”
	4.8	“For children, if it only looks slightly different, they won’t recognize it”
Concerns	4.9	“It should not be the intention that children/parents keep track of their nutritional intake on a daily basis, as this would put too much focus on it”
	4.10	“You make less contact with the patient when using a tablet or a smartphone”

##### Parents and caregivers

Closely related to the autonomy of children is the role of parents or caregivers. This role becomes less significant when children become older, and the responsibility of assessment methods shifts from parent to child. Nevertheless, “whatever age a child might have, while he/she lives at home, it will often be the parents or caregivers who do the groceries. So, you will always need them.” In some cases, PDs chose specifically to exclude caregivers during dietary assessments (quote 3.6 in Table 4). On the contrary, in the case of children with severe diseases, PDs exclusively rely on the parents or caregivers because they “don’t want to burden the children too much.”

##### Cultural background

The cultural background of the children and their caregivers affects dietary behavior assessments. When asked *about barriers for the success of an assessment method*, PDs frequently mentioned reading and writing competence of the Dutch language as well as the level of education of both child and caregiver (quote 3.7 in Table 4). PDs also assume that cultural background influences the acceptability of new technologies for dietary behavior assessments (quote 3.8 in Table 4)*.*

##### PDs

The role of PDs when it comes to dietary assessment methods is described as “to uncover everything and to keep asking if you notice anything is wrong” and subsequently, to act upon a situation. What facilitates this, is the experience of the PD in the field together with a level of mutual trust between PD and child (quote 3.9 in Table 4). Finally, PDs mention nonverbal communication as a facilitator to complete dietary behavior assessments (quote 3.10 in Table 4). All PDs had a positive attitude toward the potential use of technologies in their daily practice because “if you look at this generation, children are glued to their mobile phones. So, I think it would be valuable to let them do something in their phones or to include the phone because they are already attached to it. So, it will be more appealing to them.”

#### Domain 4: future methods

The domain *future methods* addresses the themes that emerged when future DBA technologies were discussed. This includes criteria that future methods should meet to succeed, accessible, stimulating, functional, as well as foreseen challenges and concerns. Themes and supporting quotes are presented in Table 5.

##### Accessible

When PDs were asked what they thought were important criteria for future DBA technologies to meet, PDs often mentioned criteria that support the accessibility of a technology such as easy to use, intuitive, and customer friendly. PDs mentioned different practical ideas to increase the accessibility of technological DBAs, such as using understandable terms for foods and portion sizes, using images or pictures, prompting assisting questions (quote 4.1 in Table 5), and for the technology to be available in different languages. More futuristic approaches to increase accessibility were also thought of, such as “making pictures and that it will tell you automatically ‘these were your macronutrients.’” PDs did not think that using a mobile phone for DBA would impair accessibility (quote 4.2 in Table 5).

##### Stimulating

In addition to the method being accessible for children, PDs mention that future DBA methods should be stimulating to children by being engaging, fun, and challenging. Ideas given by the PDs to engage children and make DBAs more appealing were, again, using images and pictures, providing a feeling of autonomy and insight (quote 4.3 in Table 5) as well as implementing positive feedback (quote 4.4 in Table 5). Color, point, or smiley systems were frequently pointed out as appropriate measures to provide feedback with: “I would very much like to see a sort of color system or a sort of point system, so that we don’t talk in calories but in points or in colors. That children can see, oh, maybe it’s not the best choice or maybe it’s a very good choice. And that way, they can get some insight into what they’re doing.”

##### Functional

PDs indicated that it is important that new technologies add to their personalized guidance, for instance, by providing practical and useful information (quote 4.5 in Table 5). However, what this practical information might look like differs between PDs and the goal for which the method is used, such as information on proper portion sizes, eating frequency, time spent eating (quote 4.6 in Table 5), or specific nutrients and product information. Others highlight the importance of getting an overall picture. A practical and reoccurring suggestion made by PDs to increase functionality was “linking of the app to the electronic patient file.” All PDs were clear about 1 thing: “It would be so nice to make it a little easier … that you just do it a bit quicker to get to work on your advice.”

##### Challenges

PDs felt that money and time were the biggest challenges in the implementation of new DBA technology. Other mentioned challenges included keeping children engaged and motivated (quote 4.7 in Table 5), limited cognitive abilities of children (quote 4.8 in Table 5) and, keeping applications up-to-date with the changing availability of food products.

##### Concerns

Concerns raised by PDs was DBA technology might put too much focus on dietary assessment (quote 4.9 in Table 5), and that the technology should not become too profound or controlling. To illustrate, when the idea of a tool for measuring food intake using automatically taken pictures was proposed PDs responded with [32]: “I don’t know if we should go there, to get everything mapped out. It goes a bit far, right?” and “They should not see you as a police officer who comes and checks you … with those pictures that you go there.” Moreover, the concern that technology would interfere in the contact between PD and patient was raised (quote 4.10 in Table 5).

### Quantitative results

**FIGURE 1:**
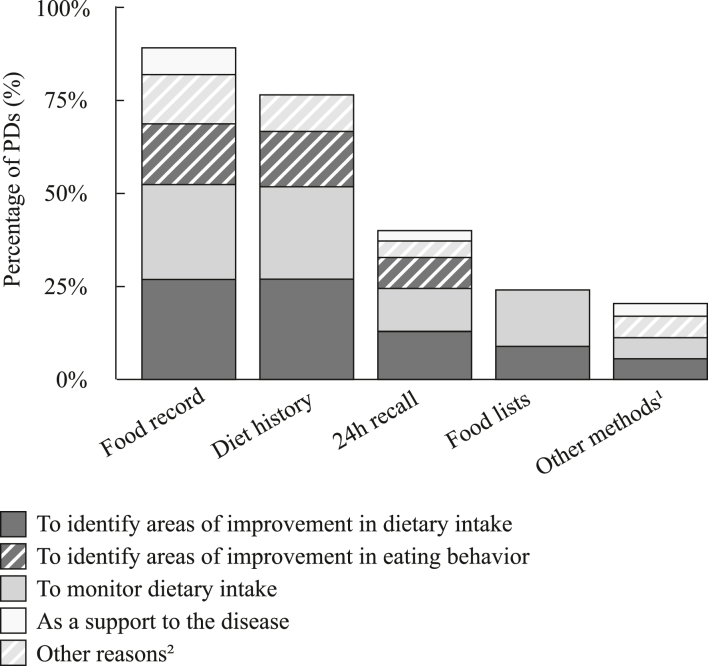
**Reported traditional methods used by pediatric dieticians for assessing dietary behavior and reasons for using them**. Each pediatric dietician was asked to select 3 methods which were most frequently used. ^1^Other methods mentioned were a combination of mentioned methods, clinical records, and fluid balances. ^2^Other reasons mentioned were a combination of mentioned reasons, to identify allergies and to create awareness of dietary behaviors. DBA, dietary behavior assessment; PD, pediatric dieticians.

The majority of PDs participating in the online survey used paper-based food records (87%) and oral diet history (74%) for DBAs (Figure 1). Reasons for use did not differ between methods (*P* = 0.51) and use of methods did not differ between health care setting, age, or years of experience (*P* > 0.73). All PDs indicated to regularly use some form of technology to support their assessments; 16 different apps, software programs, or technological tools were described. The most commonly used technology (61%) was “*Mijn Eetmeter*,” a digital food record developed by The Netherlands Nutrition Centre, followed by cameras on mobile phones to record photos (23%) and videos (26%).

**FIGURE 2:**
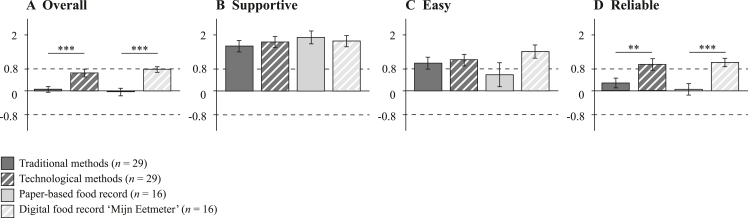
**The UEQ scores of all traditional methods averaged compared with all technological methods averaged (n = 29) and scores of the paper-based food record compared with the digital food record app “*Mijn Eetmeter*” (n = 16)**. Mean ± standard error of the UEQ score for the average of 10 qualities overall (A) and the 3 most important qualities that assessment methods should fulfill according to PDs: supportive (B), easy (C), and reliable (D). A UEQ score between −0.8 and 0.8 represents a neutral evaluation, above 0.8 a positive evaluation, and below −0.8 a negative evaluation of usability [31]. Differences in mean UEQ scores were determined with a t-test, significant differences are indicated by ∗∗ for P < 0.01 and ∗∗∗ for P < 0.001. DBA, dietary behavior assessment; PD, pediatric dietician; UEQ, user experience questionnaire.

On average, technological methods (*M* = 0.64, SD = 0.14) received higher overall UEQ scores compared with traditional methods (*M* = 0.05, SD = 0.10), *t* (28) = 4.0, *P* < 0.001 (Figure 2). Following this tendency, paper-based food record (*M* = −0.02, SD = 0.13) scored lower than its digital equivalent “*Mijn Eetmeter*” (*M* = 0.77, SD = 0.43), *t* (16) = 6.7, *P* < 0.001. *Supportive* (65%), *easy* (65%), and *reliable* (58%) were the most commonly selected quality criteria for current and future DBA when asked to select a total of 3 items. *Efficient*, *interesting*, and *leading-edge* were not selected by any PD. Both traditional and technological methods were assessed being supportive and easy to use, with no significant difference (*P* > 0.10). Discrepancy shows for the reliability of technological and traditional methods (*P* = 0.003).

**FIGURE 3:**
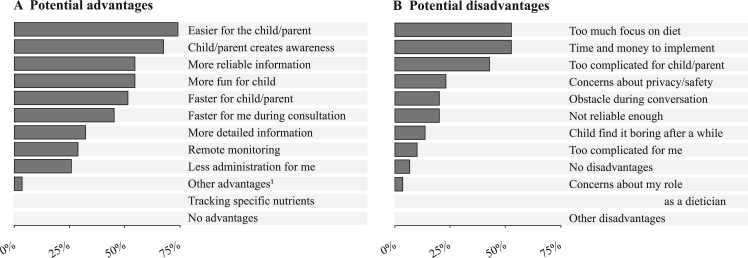
**Potential advantages (A) and disadvantages (B) of using technology for dietary behavior assessments as rated by the pediatric dieticians participating in the survey (N = 31)**. Pediatric dieticians were allowed to select as many answers as they thought were applicable. ^1^Another advantage mentioned was the potential to customize technology to specific needs.

Regarding future methods, PDs (74%) expect DBA to become easier for both child and parents when incorporating technology (Figure 3). Additionally, PDs see the potential of technology to create awareness of one's own eating behavior (68%), provide more reliable information (55%), and be more fun and engaging to children (55%). However, PDs anticipate certain disadvantages associated with DBA technology, such as an excessive emphasis on the dietary assessment (52%) and the money and time implementation will cost (52%). Foreseen advantages and disadvantages did not differ between health care settings, age, or years of experience (*P* > 0.16).

## Discussion

This mixed-method study among Dutch PDs highlighted various considerations, strengths, and weaknesses on the use of traditional DBA methods and potential newly developed DBA technology for future use. Overall, the interviewed PDs felt that traditional methods were sufficient to reach the desired goals. However, PDs also felt that the time needed to register dietary intake behaviors as well as the reliability of traditional methods can be improved, and that technology can play a role here. UEQ scores from the survey reflect this moderate appreciation of traditional methods and reveal the importance and potential of technology regarding reliability. Moreover, both PDs in the interviews and the survey foresee that future technological assessment methods increase the ease of use and have the potential to be more engaging for children. Accordingly, the overall attitude of PDs regarding the use of technology for DBAs was positive.

Our results are in agreement with Hutchesson et al. [33] who indicated that dietetics practitioners perceive a burden on the user and limited time available as major barriers in applying dietary assessments. A qualitative study by Bonilla et al. [17] showed that dieticians believe that electronic dietary assessment tools could improve the assessment quality for adults. Assessment quality is believed to be improved by increased motivation and ease of use of the methods and qualitative studies indeed indicate that nutritionists [34], parents [35,36], and adolescents [37,38] think digital applications are convenient and motivating. Nonetheless, so far, only technological tools incorporating image assistance have been shown to increase assessment quality [13,39]. Although parents and adolescents like to use mobile applications for health purposes [36,38], they agree with health care providers that technology should be a supplement rather than replace face-to-face interaction [17] and similarly, PDs in this study stated that nonverbal communication is important and that the role of technology should not become too profound. PDs also felt that suitable assessment tools are currently missing, whereas Bonilla did not reveal this opinion interviewing nonpediatric dieticians. This verifies the lack of a high-quality and validated tools specifically tailored for children, which is in agreement with previous findings that applications to date do not follow dietary guidelines, have moderate app quality [40,41], or are not validated properly [13].

For the development of a future DBA method, developers should understand that a “one-size-fits-all” solution is not feasible, and each assessment tool has different limitations for its users [2,35]. When selecting assessment tools, PDs consider these limitations in light of the target population, available time, aimed goal, and, consequently, the required level of accuracy. Guidelines to select appropriate dietary assessment tools in health research show similar considerations [3,4]. Age is considered an important factor when selecting an appropriate assessment method [33], especially in pediatric dietetics [3]. PDs distinguished 4 age categories related to the autonomy of children and, consequently, the role of parents that can be used as guidance to design suitable dietary assessment technologies: from newborn to 6 y olds, from 6 to 11 y, from 11 to 14 y, and from 14 y onward.

The age range from 6 to 11 y aligns with the third period of child’s cognitive development as classified by Piaget [42], and is known as the concrete operations period. This period is characterized by the understanding of the concept of conservation, referring to the concept of values remaining unchanged during visual transformation [43], which is essential in terms of the ability to estimate portion sizes and food knowledge. Moreover, children below the age of 6 y have an undeveloped short- and long-term memory span, which makes recalling consumed food items extremely difficult at this young age [44]. Research on dietary assessment in young children showed that 6- to 7-y-old children significantly outperform their 5- to 6-y-old younger peers, when asked to recall their lunch within 2 h after finishing [45]. Consequently, when designing assessment technologies for children until the age of ∼6 y, parents should be seen as the primary end user [46] and, from the age of 6 y onward, it should be clear who serves as the primary end user, child, caregiver, or both, and level of accuracy is requested to fulfill the purpose of the assessment method. When surrogate information cannot be provided by caregivers or there is a need to obtain data with high accuracy, development should focus on the objective, passive methods to be suitable in use below the age of 11 y. These objective, passive methods would also be of interest in situations where PDs prefer to minimize the burden on children and their parents in case of children with severe diseases as well as in case parents prefer to minimize their child’s screen time or interaction with technology. Examples of such are image- and video-based wearables [32,47] that automate the analysis of portion sizes and food items to provide information on dietary intake or acoustic sensors [48] and wrist movement trackers [49] when information on the timing and frequency of eating is of interest. However, doubt has been expressed by PDs toward the use of these automated technologies, especially privacy-related concerns as well as concerns related to the acceptance of these technologies within their patients and corresponding caregivers. Privacy is indeed a concern raised by parents [36] and adolescents [38] in the context of technology use for dietary assessment. It should be pointed out that these types of technologies are in a preliminary state [39] and that automated self-monitoring technologies further in development, such as continuous glucose monitoring, are well perceived and privacy-related concerns are not addressed by parents or adolescents [50]. Future research should focus on increasing the precision of these passive technologies to reach acceptable levels as well as focus on how to increase the acceptance of these technologies by the potential users, for example, by ensuring a privacy-safe environment.

The fourth period as classified by Piaget, the formal period, starts around the age of 11 is the final period of cognitive development in children. This period is characterized by the ability of applying logical rules in an abstract context, and by this time, the memory span of children reaches adult-level performance [44]. Also, research on dietary assessment in children points out the age of ∼10–11 y as the limit after which children become more capable of reporting their dietary intake on their own. Foster et al. [51] found that children of this age were able to report their dietary intake with comparable accuracy as their caregivers when using a tailored photograph-assisted food record and also Baranowski states that children up to the age of 10 y are not able to provide reasonably accurate and complete intake data without using proxy reporters [52]. Hence, the development of DBA technologies for children from 11 y onward, should focus on age-appropriate self-administrative methods. These age-appropriate self-administrative methods may also be applicable for children younger than 11 y when lower levels of accuracy are acceptable, the assessment tool has a rather educational purpose, or the parents are available to give surrogate information. In the case of the latter, one should make sure that the system is suitable for proxy-assisted response. For a method to be age appropriate, it should align with how children perceive the world to enhance its accessibility. For example, Foster mentions the ability of children to recognize textures, colors, and images of food packages despite their lacking ability to name specific foods [52]. This could be used to create recognizable food icons as well as food categories that align with the children’s perspectives to make, for example, digital food records more child friendly. Additionally, PDs mention using recognizable images, sound assistance, and prompting reminding questions of frequently forgotten foods and drinks. An example of such a tool already developed is ASA24-Kids [53]. This self-administered 24-h recall Web-application uses images for portion sizes, gives prompts for frequently forgotten foods, offers the capability to find misspelled foods, and is feasible for proxy reporting [54]. However, ASA24 is tailored to the American diet [6], a tool adapted to the Dutch language, diet and eating culture are lacking. Also, so far, ASA24 did not outperform interview-based or image-based assessments in its accuracy and also in its usability [6,35,53]. Image-based assessments and food records using a camera to capture consumed foods and drinks show promising results in reducing misreporting [13,39]. Most PDs participating in this study were familiar with the use of photo and video recordings to assist their assessments, and this direction would therefore be a logical direction for future development. To lower the burden on PDs, algorithms trained to estimate portion size and recognize food items from these images would be a welcome extension. However, as with the aforementioned passive methods, further research should increase the precision and accuracy of these algorithms [39]. In the meantime, proper image-based and voice-assisted food record or food recall application tailored to the Dutch language and eating culture would be a welcome alternative. Nevertheless, it should be borne in mind that self-administrative methods will always be sensitive to recall biases.

The last age category as discussed by PDs starts from the age of 14 y. These adolescents are believed to be kept responsible for assessing their own dietary intake. Although it is not explicitly studied whether adolescents older than 14 y recall or record their diet as accurate as adults, for diet history interviews and estimated food records, under the report when compared with the method of doubly labeled water is of a comparable level [55,56]. Teens have expressed the desire to use technology-based tools to keep track of their health [38] and communicate with their health care providers [37]. Because “lack of motivation” is reported as a challenge in this age range [7], future research should point out what factors increase compliance in this age range and how technology could incorporate these factors. Examples of such factors could be assisting customizable agents; video games; adding a narrative, appealing, and familiar interface design; rewards; social connection; and personal goal setting [38,57,58]. Additionally, attention must be given to the concern raised by PDs regarding the possibility of creating too much focus on diet when using technologies for DBA. This concern is particularly relevant in this age category due to the high incidence of eating disorders [59] and the potentially harmful side-effect of dietary self-monitoring in young women [60].

## Strength and limitations

This study is limited by selection bias; recruited PDs may show higher interest in innovative technologies for DBAs. Also, the sample size is relatively small, but the survey largely agreed with the results of the interviews. To our knowledge, this is the first study addressing the perspective of dieticians in pediatric health care settings, which provides valuable input for further developments in the field. Future research, both qualitative and quantitative, is warranted to broaden our understanding of how dietary behavior assessment methods can be tailored to the needs of children in different care situations and age categories to increase its usability. Furthermore, it is recommended to actively let PDs be part of the development of future technologies for DBA, both to use their knowledge on practical implementation and to increase acceptance of technologies in the future.

In conclusion, PDs have a positive attitude toward the use of technology for dietary behavior assessments in children and believe that technology could enhance the reliability of the assessment as well as lower the burden for child, caregiver, and dieticians. However, sufficient technologies to be used in pediatric dietetics are lacking. Further development of passive and self-administrative assessment technologies should be tailored to the needs of children in different care situations and age categories to increase its usability among children, their caregivers, and dieticians.

## Funding

The research described in this article was financially supported by a grant from the Regiodeal Foodvalley (162135) and by the 4 Dutch Technical Universities, 4TU—Pride and Prejudice program (4TU-UIT-346).

## Author disclosures

The authors report no conflicts of interest.

## Author contributions

The authors’ responsibilities were as follows—FJG, ML, EMB, and GC: designed the research; FJG: conducted research, wrote the article, and had primary responsibility for the final content; FJG, ML, and RFW: analyzed the data; and all authors: read and approved the final manuscript.

## Data availability

Data described in the manuscript, code book, and analytic code will be made available upon request.

## Appendix A Supplementary data

Multimedia component1 Multimedia component1

## References

[1] NCD Risk Factor Collaboration (NCD-RisC) (2017). Worldwide trends in body-mass index, underweight, overweight, and obesity from 1975 to 2016: a pooled analysis of 2416 population-based measurement studies in 128·9 million children, adolescents, and adults. Lancet.

[2] Brouwer-Brolsma E.M., Lucassen D., de Rijk M.G., Slotegraaf A., Perenboom C., Borgonjen K. (2020).

[3] Magarey A., Watson J., Golley R.K., Burrows T., Sutherland R., McNaughton S.A. (2011). Assessing dietary intake in children and adolescents: considerations and recommendations for obesity research. Int. J. Pediatr. Obes..

[4] Cade J.E., Warthon-Medina M., Albar S., Alwan N.A., Ness A., Roe M. (2017). DIET@NET: best practice guidelines for dietary assessment in health research. BMC Med.

[5] Dao M.C., Subar A.F., Warthon-Medina M., Cade J.E., Burrows T., Golley R.K. (2019). Dietary assessment toolkits: an overview. Public Health Nutr.

[6] Bekelman T.A., Johnson S.L., Steinberg R.I., Martin C.K., Sauder K.A., Luckett-Cole S. (2022). A qualitative analysis of the remote food photography method and the automated self-administered 24-hour dietary assessment tool for assessing children’s food intake reported by parent proxy. J. Acad. Nutr. Diet..

[7] Pérez-Rodrigo C., Escauriaza B.A., Bartrina J.A., Allúe I.P. (2015). Dietary assessment in children and adolescents: issues and recommendations. Nutr. Hosp..

[8] Walker J.L., Ardouin S., Burrows T. (2018). The validity of dietary assessment methods to accurately measure energy intake in children and adolescents who are overweight or obese: a systematic review. Eur. J. Clin. Nutr..

[9] Baxter S.D., Hardin J.W., Royer J.A., Guinn C.H., Smith A.F. (2008). Insight into the origins of intrusions (reports of uneaten food items) in children’s dietary recalls, based on data from a validation study of reporting accuracy over multiple recalls and school foodservice production records. J. Am. Diet. Assoc..

[10] Eldridge A.L., Piernas C., Illner A.K., Gibney M.J., Gurinović M.A., de Vries J.H.M. (2018). Evaluation of new technology-based tools for dietary intake assessment-an ILSI Europe dietary intake and exposure task force evaluation. Nutrients.

[11] Zhao X., Xu X., Li X., He X., Yang Y., Zhu S. (2021). Emerging trends of technology-based dietary assessment: a perspective study. Eur. J. Clin. Nutr..

[12] Cade J.E. (2017). Measuring diet in the 21st century: use of new technologies. Proc. Nutr. Soc..

[13] Kouvari M., Mamalaki E., Bathrellou E., Poulimeneas D., Yannakoulia M., Panagiotakos D.B. (2021). The validity of technology-based dietary assessment methods in childhood and adolescence: a systematic review. Crit. Rev. Food Sci. Nutr..

[14] Thabrew H., Fleming T., Hetrick S., Merry S. (2018). Co-design of eHealth interventions with children and young people. Front. Psychiatry..

[15] Nguyen T.T.H., Saranto K., Tapanainen T., Ishmatova D. (2014). A review of health information technology implementation success factors: importance of regulation and finance, 2014.

[16] Kaye R., Kokia E., Shalev V., Idar D., Chinitz D. (2010). Barriers and success factors in health information technology: a practitioner’s perspective. J. Manag. Mark. Healthc..

[17] Bonilla C., Brauer P., Royall D., Keller H., Hanning R.M., DiCenso A. (2015). Use of electronic dietary assessment tools in primary care: an interdisciplinary perspective. BMC Med. Inform. Decis. Mak..

[18] Tong A., Sainsbury P., Craig J. (2007). Consolidated criteria for reporting qualitative research (COREQ): a 32-item checklist for interviews and focus groups. Int. J. Qual. Health Care..

[19] Eysenbach G. (2004). Improving the quality of web surveys: the checklist for reporting results of internet e-surveys (CHERRIES). J. Med. Internet Res..

[20] Guest G., Bunce A., Johnson L. (2006). How many interviews are enough?: an experiment with data saturation and variability. Field Methods.

[21] Weller S.C., Vickers B., Bernard H.R., Blackburn A.M., Borgatti S., Gravlee C.C. (2018). Open-ended interview questions and saturation. PLOS ONE.

[22] Hill R. (1998). What sample size is “enough” in internet survey research. Interpers. Comput. Tech..

[23] Fisher J.D., Fisher W.A. (1992). Changing AIDS-risk behavior. Psychol. Bull..

[24] Morville P., Sullenger P. (2010). Ambient findability: libraries, serials, and the internet of things. Ser. Librarian..

[25] Janghorban R., Latifnejad Roudsari R., Taghipour A. (2014). Skype interviewing: the new generation of online synchronous interview in qualitative research. Int. J. Qual. Stud. Health Well Being.

[26] Schrepp M., Hinderks A., Thomaschewski J. (2017). Design and evaluation of a short version of the user experience questionnaire (UEQ-S). Int. J. Interact. Multimedia Artif. Intell..

[27] Qualtrics (2021). Qualtrics [Computer software].

[28] Amberscript Global B.V. (2021). Amberscript [Computer software].

[29] Provalis Research (2020). QDA Minor Lite (Version 2.0.8) [Computer software.

[30] RStudio Team (2020). RStudio: Integrated Development for R (Version 1.4.1106) [Computer software]. PBC.

[31] Schrepp M., Hinderks A., Thomaschewski J. (2014). International Conference of Design, User Experience, and Usability.

[32] Beltran A., Dadabhoy H., Ryan C., Dholakia R., Jia W., Baranowski J., Sun M., Baranowski T. (2018). Dietary assessment with a wearable camera among children: feasibility and intercoder reliability. J. Acad. Nutr. Diet..

[33] Hutchesson M., Rollo M., Burrows T., McCaffrey T.A., Kirkpatrick S.I., Kerr D. (2021). Current practice, perceived barriers and resource needs related to measurement of dietary intake, analysis and interpretation of data: a survey of Australian nutrition and dietetics practitioners and researchers. Nutr. Diet..

[34] Saronga N., Mosha I.H., Stewart S.J., Bakar S., Sunguya B.F., Burrows T.L. (2022). A mixed-method study exploring experiences and perceptions of nutritionists regarding use of an image-based dietary assessment system in Tanzania. Nutrients.

[35] Bekelman T.A., Martin C.K., Johnson S.L., Glueck D.H., Sauder K.A., Harrall K.K. (2022). A comparison of the remote food photography method and the automated self-administered 24-h dietary assessment tool for measuring full-day dietary intake among school-age children. Br. J. Nutr..

[36] Wild C.E.K., Egli V., Rawiri N.T., Willing E.J., Hofman P.L., Anderson Y.C. (2022). It’s more personal if you can have that contact with a person”: qualitative study of health information preferences of parents and caregivers of children with obesity in New Zealand. Health Soc. Care Community..

[37] Radovic A., McCarty C.A., Katzman K., Richardson L.P. (2018). Adolescents’ perspectives on using technology for health: qualitative study. JMIR Pediatr. Parent..

[38] San Giovanni C.B., Dawley E., Pope C., Steffen M., Roberts J. (2021). The doctor will “friend” you now: a qualitative study on adolescents’ preferences for weight management app features. South. Med. J..

[39] Höchsmann C., Martin C.K. (2020). Review of the validity and feasibility of image-assisted methods for dietary assessment. Int. J. Obes. (Lond)..

[40] Brown J.M., Franco-Arellano B., Froome H., Siddiqi A., Mahmood A., Arcand J. (2022). The content, quality, and behavior change techniques in nutrition-themed mobile apps for children in Canada: app review and evaluation study. JMIR MHealth UHealth.

[41] Schoeppe S., Alley S., Rebar A.L., Hayman M., Bray N.A., Van Lippevelde W. (2017). Apps to improve diet, physical activity and sedentary behaviour in children and adolescents: a review of quality, features and behaviour change techniques. Int. J. Behav. Nutr. Phys. Act..

[42] Beilin H., Fireman G. (1999). The foundation of Piaget’s theories: mental and physical action. Adv. Child Dev. Behav..

[43] Viarouge A., Houdé O., Borst G. (2019). The progressive 6-year-old conserver: numerical saliency and sensitivity as core mechanisms of numerical abstraction in a Piaget-like estimation task. Cognition.

[44] Gathercole S.E. (1998). The development of memory. J. Child Psychol. Psychiatry..

[45] Warren J.M., Henry C.J., Livingstone M.B., Lightowler H.J., Bradshaw S.M., Perwaiz S. (2003). How well do children aged 5–7 years recall food eaten at school lunch?. Public Health Nutr.

[46] Fialkowski M.K., Kai J., Young C., Langfelder G., Ng-Osorio J., Shao Z. (2022). An active image-based mobile food record is feasible for capturing eating occasions among infants ages 3–12 months old in Hawai’i. Nutrients.

[47] Farooq M., Doulah A., Parton J., McCrory M.A., Higgins J.A., Sazonov E. (2019). Validation of sensor-based food intake detection by multicamera video observation in an unconstrained environment. Nutrients.

[48] Makeyev O., Lopez-Meyer P., Schuckers S., Besio W., Sazonov E. (2012). Automatic food intake detection based on swallowing sounds. Biomed. Signal Process. Control..

[49] Shen Y., Salley J., Muth E., Hoover A. (2017). Assessing the accuracy of a wrist motion tracking method for counting bites across demographic and food variables. IEEE J. Biomed. Health Inform..

[50] Lawton J., Blackburn M., Allen J., Campbell F., Elleri D., Leelarathna L. (2018). Patients’ and caregivers’ experiences of using continuous glucose monitoring to support diabetes self-management: qualitative study. BMC Endocr. Disord..

[51] Foster E., Hawkins A., Barton K.L., Stamp E., Matthews J.N., Adamson A.J. (2017). Development of food photographs for use with children aged 18 months to 16 years: comparison against weighed food diaries –the Young Person’s Food Atlas (UK). PLOS ONE.

[52] National Academies of Sciences (2022).

[53] Diep C.S., Hingle M., Chen T.-A., Dadabhoy H.R., Beltran A., Baranowski J. (2015). The automated self-administered 24-hour dietary recall for children, 2012 version, for youth aged 9 to 11 years: a validation study. J. Acad. Nutr. Diet..

[54] Sharpe I., Kirkpatrick S.I., Smith B.T., Keown-Stoneman C.D.G., Omand J., Vanderhout S. (2021). Automated self-administered 24-h dietary assessment tool (ASA24) recalls for parent proxy-reporting of children’s intake (>4 years of age): a feasibility study. Pilot Feasibility Stud.

[55] Burrows T.L., Martin R.J., Collins C.E. (2010). A systematic review of the validity of dietary assessment methods in children when compared with the method of doubly labeled water. J. Am. Diet. Assoc..

[56] Burrows T.L., Ho Y.Y., Rollo M.E., Collins C.E. (2019). Validity of dietary assessment methods when compared to the method of doubly labeled water: a systematic review in adults. Front. Endocrinol..

[57] Lu A.S., Baranowski J., Islam N., Baranowski T. (2014). How to engage children in self-administered dietary assessment programmes?. J. Hum. Nutr. Diet..

[58] Chan A., Kow R., Cheng J.K. (2017). Adolescents’ perceptions on smartphone applications (apps) for health management. J. Mobile Tech. Med..

[59] van Son G.E., van Hoeken D., Bartelds A.I., van Furth E.F., Hoek H.W. (2006). Time trends in the incidence of eating disorders: a primary care study in the Netherlands. Int. J. Eat. Disord.

[60] Hahn S.L., Linxwiler A.N., Huynh T., Rose K.L., Bauer K.W., Sonneville K.R. (2021). Impacts of dietary self-monitoring via MyFitnessPal to undergraduate women: a qualitative study. Body Image.

