# Supplementary material for: Dietary Behavior Assessments in Children—A Mixed-Method Research Exploring the Perspective of Pediatric Dieticians on Innovative Technologies
Source: Curr Dev Nutr. 2023 Apr 26;7(6):100091. doi: 10.1016/j.cdnut.2023.100091 (PMC10196961; doi:10.1016/j.cdnut.2023.100091)
Supplement: Multimedia component1 [file mmc1.docx]

**Supplementary data ‘Dietary behaviour assessments in children – a mixed-method research exploring the perspective of paediatric dietitians on innovative technologies’**

**Femke J. de Gooijer**

**Supplementary Table 1. Interview guide based on the Information-motivation-behaviour model (23) and the User Experience Honeycomb model (24).**

| **GENERAL** | |
| --- | --- |
| Age, function, work experience;  What do you think are positive and negative aspects of your job? | |
| **USER** | |
| **Knowledge** | Which methods for dietary behaviour assessment (DBA) do you know?  Which technical tools do you know? |
| **Skills** | Which methods for DBA do you use on daily use regularly?  Which technical tools do you use regularly? |
| **Attitude** | What do you think of current methods for DBA?  What do you think of current technological methods DBA? |
| **PREVIOUS EXPERIENCES** | |
| **Useful** | For what purpose do you use current methods for DBA in children?  Do you find that current methods help you sufficiently achieve this goal? |
| **Usable** | What do you find easy to use in current methods for DBA in children?  What do you find limitations in using current methods DBA in children? |
| **Credible** | What do you think of the reliability of current methods?  What do you think of the accuracy of current methods?  Are there ethical considerations involved in using current methods? |
| **Accessible** | What is your experience with children's willingness in using methods for DBA?  What do you perceive to be factors that influence this willingness?  To what extent do children's skills influence the methods for DBA?  What are factors that influence these skills?  Are technological tools for DBA accessible to all of your clients?  How do you feel about the amount of time that current methods take? |
| **FUTURE EXPERIENCES** | |
| **Useful** | Do you think that technical tools for DBA in children add anything to your current way of working?  If so, what technical tools would be valuable? |
| **Usable** | How do you envision the use of such technologies?  What do you think are elements that contribute to the success of a new technology?  What do you foresee as potential obstacles in the use of technology? |
| **Credible** | How do you view the reliability of new technological tools?  How do you view the accuracy of new technological tools?  What ethical considerations are involved in the use of new technological tools? |
| **Accessible** | Do you think new technological tools increase or decrease children's accessibility to DBA? |
| Do you think that new technological tools increase or decrease children's willingness for DBA? | |
| **ENDING** | |
| Do you have any further comments on conducting DBA in children or new technological developments in this area? | |

**
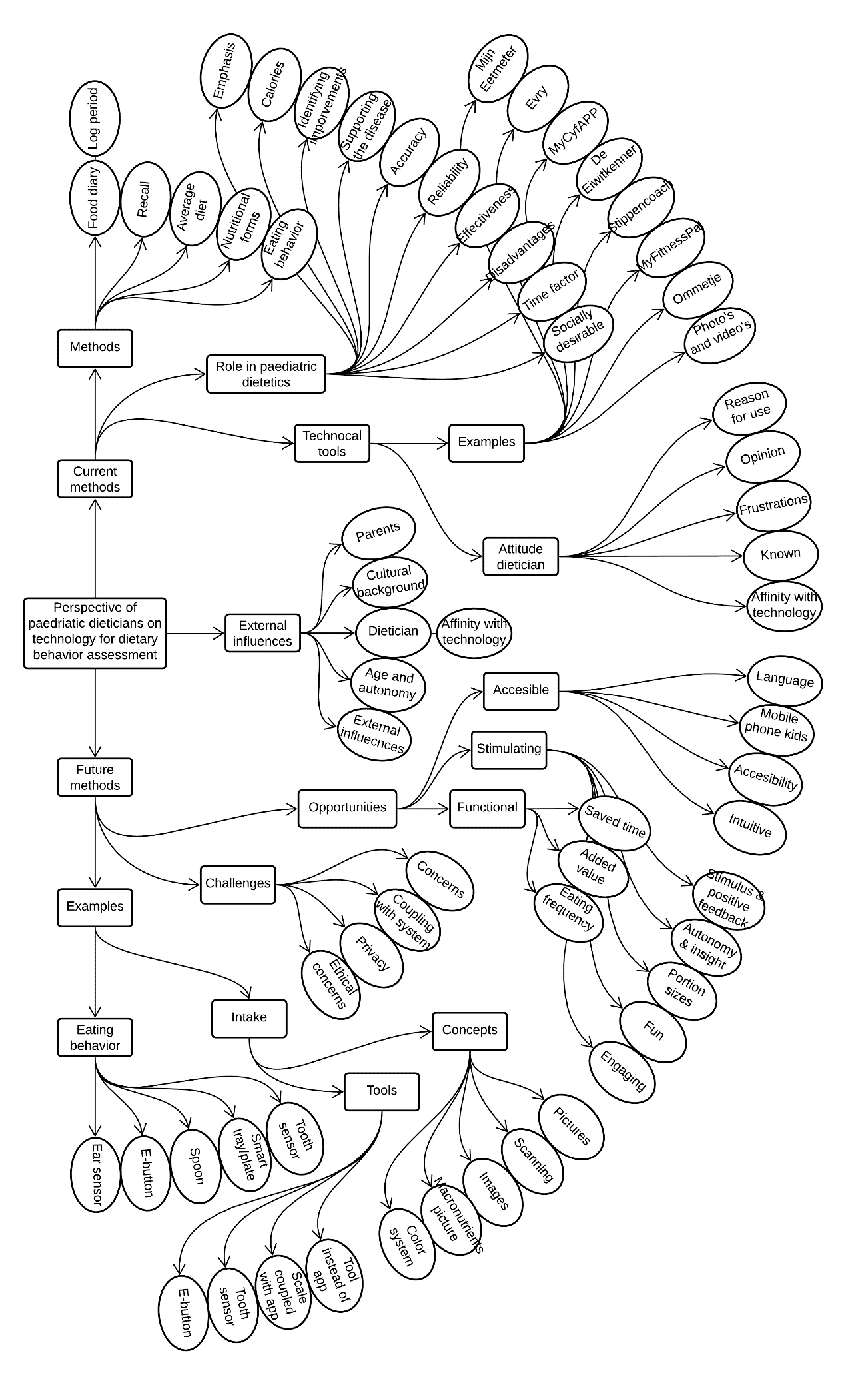
**

**Supplementary Figure 1. Coding tree emerged from the interviews with paediatric dietitians on innovative technologies for dietary behaviour assessment in children.**
